# Clinical characterization and molecular analysis of X-linked juvenile retinoschisis in a northern Chinese cohort

**DOI:** 10.3389/fgene.2026.1796743

**Published:** 2026-05-14

**Authors:** Huihui Sun, Jindou Shi, Jiancang Wang, Suling Yang, Xiexie Liu, Peiyao Jin, Zheng Zheng

**Affiliations:** 1 Department of Ophthalmology, The Children’s Hospital of Hebei Province, Hebei Provincial Clinical Research Center for Child Health and Disease, Shijiazhuang, China; 2 Department of Imaging Department Hebei General Hospital, Hebei Medical University, Shijiazhuang, China; 3 Department of Ophthalmology, Nanjing Medical University Eye Hospital, Nanjing, China; 4 Department of Ophthalmology, Shanghai General Hospital, Shanghai Jiao Tong University School of Medicine, Shanghai, China; 5 National Clinical Research Center for Eye Diseases, Shanghai, China

**Keywords:** gene mutation, gene therapy, genetic-phenotypicvariability, retinoschisin 1, X-linked juvenile retinoschisis

## Abstract

**Purpose:**

This study aims to explore the clinical features and genetic findings associated with X-linked juvenile retinoschisis (XLRS) in affected patients.

**Methods:**

This study included 16 patients with XLRS from 13 unrelated families between 2016 and 2024. Genomic DNA from peripheral blood leukocytes of the probands were subjected to whole-exome sequencing or direct sequence. Comprehensive analyses of molecular genetic profiles and detailed ophthalmic evaluations were performed.

**Results:**

We identified 12 retinoschisin 1 (*RS1*) variants, and five of them were novel. Missense variants (7/9, 75%) in the discoidin domain were the most common mutations. All 16 patients aged 4–45 years old were males, and the mean visual acuity was 0.58 ± 0.49 (Log MAR). The funduscopic observations were consistent with typical XLRS presentations. Notably, distinctive clustered pigmentation exhibited 75% penetrance (3/4 cases) in individuals harboring the novel Q43* mutation. Optical coherence tomography revealed macular schisis in 27 eyes (87.1%), peripheral schisis in six eyes (19.35%), and retinal atrophic changes in five eyes (16.13%). Schisis predominantly affected the inner nuclear layer (27/31, 87.10%). Additionally, other less common abnormalities included asymmetric schisis in patients with the R182C variant, presenting with schisis in one eye and a relatively normal fellow eye. Furthermore, two individuals with the Q43* nonsense mutation exhibited mild electroretinogram abnormalities with a preserved b-to-a amplitude ratio. Given these findings, larger multicenter studies are warranted to validate the observed associations.

**Conclusion:**

This study comprehensively analyzed the genetic and clinical features of XLRS in a northern Chinese cohort. Five novel variants were identified, expanding the known mutational spectrum and enriching the clinical manifestation. Distinct pigment clusters (Q43*) and asymmetric schisis (R182C) appeared consistently within our limited cohort carrying specific mutations, which may potentially facilitate the diagnosis of XLRS.

## Introduction

X-linked juvenile retinoschisis (XLRS, OMIM 312700), first described in 1898, is a rare X-linked recessive inherited retinal degenerative disease predominantly affecting men, with a prevalence rate between 1:5000 and 1:25,000 (Ambrosio et al., 2019; Ambrosio et al., 2021; J, 1898). XLRS results from a mutation in the retinoschisis 1 (*RS1*) gene, which comprises six exons and encodes a 224 amino-acid protein termed retinoschism (RS) (Sauer et al., 1997; Warneke-Wittstock et al., 1998). RS comprises a 23 amino acid N-terminal leader, a dominant 157 amino acid discoidin domain, a 39 amino acid Rs1 domain upstream of the discoidin domain, and a five amino acid C-terminal segment (Molday et al., 2012). RS is highly expressed in retinal photoreceptors and bipolar cells, and it is essential in maintaining the structural integrity of the retina (Honig and Shapiro, 2020; Hoon et al., 2014).

XLRS-affected males are often diagnosed through visual impairments discovered during school screening (Chunjie Chen et al., 2020). In the majority of patients, visual impairments deteriorate progressively with age. However, complications like vitreous hemorrhage and retinal detachment can cause vision to get worse very quickly (Huang et al., 2020). A car wheel-like pattern is characteristically observed in the patient’s fovea, with macular or peripheral schisis detectable via optical coherence tomography (OCT). Additionally, full-field electroretinogram (ERG) findings usually show that a relative preservation of a-wave amplitude and a substantial reduction in the dark-adapted b-wave amplitude, indicating dysfunction of inner retinal cells. Combining clinical examinations and molecular-genetic testing is recommended to confirm the precise diagnosis.

To date, over 700 unique variants in *RS1* have been reported, predominantly consisting of missense mutations located within the discoidin domain. Significant variation in the clinical presentation of XLRS exists even among members of the same family, despite its complete penetrance. Previous clinical studies evaluating the correlation between variants and clinical outcomes remain unclear. However, as a monogenic, relatively common, and severe variant of retinal dystrophy, XLRS remains a compelling target for gene therapeutic intervention. Significant progress has recently been made in developing gene therapies for XLRS. Phase I/II human clinical trials have been conducted and are presently ongoing. Nevertheless, the outcomes remain unsatisfactory. Therefore, a comprehensive understanding of the clinical features, phenotypic variability, and long-term natural progression of inherited retinopathies such as XLRS is essential for establishing optimal eligibility criteria and endpoints for future gene therapy trials.

This study aimed to analyze and document the clinical features of XLRS in a cohort of patients from northern China with molecularly confirmed *RS1* mutations.

## Materials and methods

### Clinical data

**FIGURE 1:**
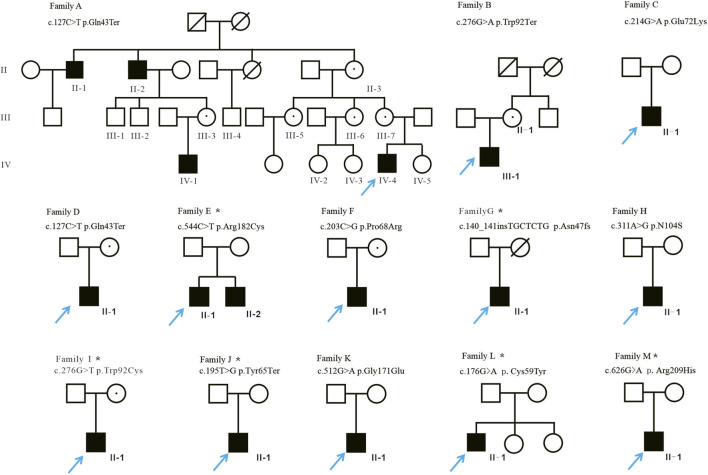
Identification of *RS1* gene mutations. Pedigrees of 13 Chinese families with X-linked retinoschisis and segregation of the identified *RS1* mutations. Affected males are represented by black squares. Only genetically tested females and obligate carriers are indicated as a circle with a dot. The asterisk indicates the pedigrees that underwent next-generation sequencing (NGS).

This study recruited 13 probands from the ophthalmology department of the Children’s Hospital of Hebei Province between 2016 and 2024. Each proband underwent a detailed family history collection, and all available family members were included where possible (Figure 1). All participants underwent comprehensive ophthalmic examinations, including best corrected visual acuity (BCVA), slit lamp biomicroscopy, cycloplegic refraction, fundus photography performed with a Hotline retinal camera (Gaoshi Medical Equipment Co., Shenzhen, China), OCT acquired using a Spectral-Domain OCT system (Spec-TR-04852, Heidelberg Engineering GmbH, Heidelberg, Germany), and full-field ERG recorded with a ROLAND CONSULT RETI-PORT/SCAN 21 system (Roland Consult GmbH, Brandenburg, Germany) in accordance with ISCEV standards (Robson et al., 2022),with the detailed acquisition parameters, imaging protocols, and quality-control procedures provided in the Supplemental Methods. Because of retinal detachment (RD), we could not get clinical data from Family A II-1’s right eye. All examinations were performed by experienced ophthalmologists (Table 1).

**TABLE 1:** Clinical characteristics of individuals with XLRS in Chinese families.

Family number	Pedigree	Age	First symptom	BCVA	Diopter	FP	Macular schisis	Perigeral schisis	ERG	Complications
Family A*	II-2	45	Poor sight	LP/0.2	NA	Atrophy of the fovea in the left eye	NA/+	NA/+	Negative ERG	Retinal atrophy in the left eye; cataract, RD, VRH in the right eye
​	IV-1	4	Vision impairment	0.8/0.8	OD:+0.25D OS:+0.50D/-0.50D*168	Cystic edema of the macular fovea	+/+	−/−	The decreasing amplitude of b wave	No special
​	IV-4	9	Vision impairment	0.6/0.8	OD:+1.25D/-0.50D*180 OS:+1.00D/-0.50D*180	Macular edema	+/+	−/−	The decreasing amplitude of a and b waves	No special
Family B*	III-1	10	Nystagmus	0.3/0.3	OD:−2.25D/-1.25D*176 OS:−1.00D/-0.50D*85	Cystic edema of the macular	+/+	−/−	The decreasing amplitude of a and b waves	No special
Family C	II-1	7	Vision impairment	0.6/0.6	OD:+1.25D OS:+1.25D	Macular edema, spoke-wheel pattern	+/+	−/−	The decreasing amplitude of a and b waves	No special
Family D*	II-1	7	Low vision	0.4/0.2	OD:+2.00D/-1.50D*15 OS:+2.00D/-1.50D*175	Cystic edema of the macular fovea	+/+	−/−	NA	No special
Family E	II-1	15	Exotropia	0.4/0.02	OD:+0.50D/-1.00D*10 = 0.6 OS:+0.50D/+1.0D*80 = 0.02	Pigment changes in both eyes, radiating macular in the left eye	−/+	+/+	The decreasing amplitude of a and b waves	No special
​	II-2	15	Exotropia	FC/1m /0.6	OD:+6.00D/+1.00D*10 OS:+0.50D/+1	The pigmentary changes, vascular sheathing in the right eye. Nearly normal in the left eye	+/−	+/−	The decreasing amplitude of a and b waves	No special
Family F	II-1	11	low vision	0.4/0.6	OD:+1.00D/-0.50D*35 OS:+1.00D/-0.50D*165	Spoke-wheel pattern	+/+	−/−	The decreasing amplitude of a and b waves	No special
Family G*	II-1	14	low vision	0.6/0.4	OD:−1.50D/-0.50D*85 OS:−4.0D/-0.50D*160	Macular edema	+/+	−/−	The decreasing amplitude of a and b waves	No special
Family H	II-1	16	Low vision	0.2/0.4	OD:+5.50D/+1.25D*63 OS:+8.0D	Spoke-wheel pattern	+/+	−/−	NA	No special
Family I	II-1	9	Nystagmus	0.15/0.3	OD:−0.75D/-1.00D*90 OS:−0.25D/-0.75D*75	Macular edema	+/+	−/−	NA	No special
Family J*	II-1	23	Poor sight	0.2/0.3	OD:+0.25D/-0.75D*85 OS:−0.50D/+1.25D*90	Hyperpigmented deposits vascular sheathing retinal atrophy	−/−	−/−	Negative ERG	Retinal atrophy of both eyes
Family K	II-1	9	Poor sight	0.4/FC/50 cm	OD:+0.75D/-1.25D*176 = 0.4	RD of the left eye, hyperpigmented deposits	+/+	+/+	NA	No special
Family L	II-1	11	Poor sight	0.08/0.25	OD:+5.0D/+1.00D*13 = 0.12 OS:+1.0D/+1.75D*76 = 0.03	Attenuated retinal vessels, hyperpigmented deposits, and retinal atrophy	+/+	+/+	The decreasing amplitude of a and b waves	RD of the right eye
Family M	II-1	8	Poor sight	0.3/0.3	OD:+4.75D/+2.50D*90 OS:+5.5D/+3.0D*95	Macular edema	+/+	−/−	The decreasing amplitude of b wave	No special

No, number; * stands group B including nonsense or insert mutations. OD, right eye; OS, left eye; OU, binocular; DS, spherical diopter; DC, cylinder diopter; BCVA, Best-corrected visual acuity; NA, not available; RPE, retinal pigment epithelium; ERG, electroretinogram; OCT, optical coherence tomography; RD, retinal detachment; VRH, vitreous hemorrhage.

### Genetic detection and mutation assessment

Genetic analysis was conducted using a combined approach, including whole-exome sequencing (WES) and Sanger sequence. Sanger sequence was used to detect the genetic mutation variants of *RS1* of families A, B, C, D, F, H, and K. Families E, G, I, J, L, and M were detected by WES (including genes involved in common inherited eye diseases).was conducted by Fujun Biotechnology (Fuzhou, China), an accredited commercial genomics facility. Genomic DNA was extracted from the peripheral blood. All six exons of the *RS1* gene were screened using primers detailed in Supplementary Table S1. Polymerase chain reaction amplicons (ABI Veriti, United States) and the generated products were verified as normal and subsequently sequenced. Additionally, the collected peripheral blood samples were screened by NGS. Variant interpretation followed the American College of Medical Genetics and Genomics guidelines (Richards et al., 2015). And all variants were submitted into ClinVar (SUB15743707, 11/3/2025), as well as accession numbers are provided throughout the text (Supplementary Table S2).

### Statistical methods

Statistical Package for the Social Sciences software for Windows (Version 22.0, IBM Corp.) was utilized for statistical analysis. BCVA was converted to the logarithm of the minimum angle of resolution (logMAR). LogMAR values of 0, 1.0, 2.0, and 3.0 corresponded to Snellen visual acuity measurements of 1.0, 0.1, counting fingers, and hand movement, respectively. Patients with light perception or no light perception were excluded from the analysis (Lange et al., 2008; Schulze-Bonsel et al., 2006). The Spearman correlation was used to assess the association between BCVA and age in all patients, in group A patients who harbored missense mutations, and in group B patients who harbored nonsense or insert mutations. Continuous data are presented as mean ± SD with 95% confidence intervals (CI). Fisher’s exact test was used for categorical comparisons in the 2 × 2 table method. A *P* < 0.05 was considered statistically significant.

## Results

### Genetic characteristics

**FIGURE 2:**
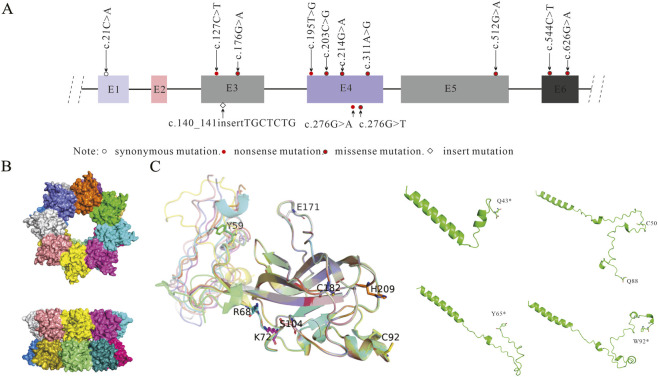
Locations of variants within retinoschisin structure. **(A)** Schematic drawing of the *RS1* gene with the six exons represented by boxes and introns represented as connecting lines between the exons. The variants are marked by the symbols illustrated in the legend. **(B)** Top and side view of a retinoschisin double octamer containing 16 subunits marked in colors. **(C)** Locations of detected missense variants in a 3-dimensional model of a mature retinoschisin subunit, and specific nonsense mutations including Q43*, W50Cfs*38, Y65*, and W92* were also displayed in a model of the truncated retinoschisin structure.

A total of 16 patients with XLRS from 13 unrelated Chinese families were recruited, and genetic diagnoses were successfully conducted (Figure 1). In total, 12 distinct *RS1* variants were identified, including five novel mutations: c.127C>T, c.140141inserTGCTCTG, c.195T>G, c.512G>A, and c.544C>T. Supplementary Table S2 presents the potential pathogenicity of these variants. Three types mutations were identified, including eight missenses c.176G>A, c.203C>G, c.214G>A, c.276G>T, c.311A>G, c.512G>A, c.544C>T, c.626G>A, 66.67%, nonsense mutations (c.127C>T, c.195T>G, c.276G>A, and one frameshift (c.140-141inserTGCTCTG, 8.33%). Families A and D harbored the identical mutation (Q43*). Most variants (75.0%, n = 9) were located in the discoidin domain, which is encoded by exons 4 (50.0%, n = 6), 5 (8.33%, n = 1), and 6 (16.67%, n = 2) (Figure 2).

### Clinical features

#### Disease presentation

All participants were male, with bilateral XLRS observed in 13 patients and unilateral involvement in two patients (Families E II-1 and II-2). At presentation, self-reported symptoms included poor vision (75.0%, n = 12), nystagmus (12.5%, n = 2), and exotropia (12.5%, n = 2). Among those with low vision, two patients had a history of retinal detachment. The mean age of the patients was 11.2 ± 4.7 years (range: 4–45 years; 95% CI: 8.20–18.43) with one outlier aged 45 years. The mean BCVA was 0.58 ± 0.49 logMAR (range: finger counting to 20/25; 95% CI: 0.40–0.76). Stratified by age, patients older than 10 years had a mean BCVA of 0.66 ± 0.49 logMAR (n = 17 eyes; 95% CI: 0.40–0.92), while those younger than 10 years had a mean BCVA of 0.48 ± 0.49 logMAR (n = 14 eyes; 95% CI: 0.20–0.77) without statistically significant difference (*P* = 0.331). The mean spherical equivalent among all participants was 1.44 ± 2.93 diopters (DS) (range: +8.0DS to −4.25 DS; n = 29 eyes; 95% CI: 0.31–2.57). Among these, six eyes exhibited myopia, with an average refractive error of −2.0 ± 1.33 DS (range: −0.625 to −4.0 DS; 95% CI: −3.40 to −0.599), while 19 eyes demonstrated hyperopia, with a refractive range of 2.88 ± 2.63 DS (range: +0.75 to +8.0 DS, 95% CI: 1.56–4.09).

#### Structure and functional outcomes of the XLRS cohort

**FIGURE 3:**
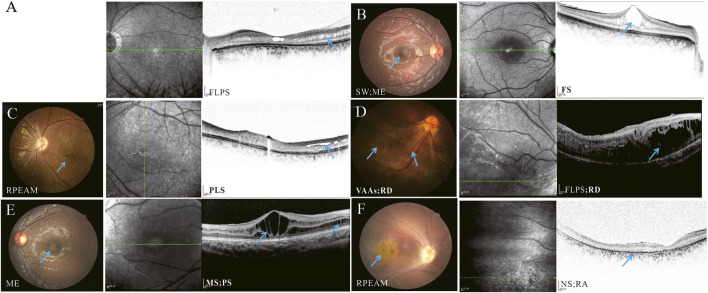
Representative fundus photographs and optical coherence tomography (OCT) images depicting different types of retinoschisis. **(A)** Direct fundus of Family A II-2 indicates pale optic disc and distinctively complete atrophy of retina (data not indicated). OCT findings of patients with foveal schisis plus peripheral schisis and macular atrophy. **(B)** Patient (Family F II-1) has a spoke-wheel pattern and edema in the macular. In fundus photographs, OCT indicates foveal schisis only. **(C)** Fundus photographs of the patient (Family E II-1) indicate pigment changes in the macular, and OCT indicates peripheral lamellar schisis. **(D)** Fundus of patient (Family L II-1) indicates retinal vascular rigidity, hyperpigmented deposits, and retinal detachment in the inferotemporal region. OCT indicates macular and peripheral schisis as well as retinal detachment. **(E)** Fundus photographs of the patient (Family M II-1) indicates macular edema, and OCT indicates foveal and peripheral schisis. **(F)** Fundus of patients (Family J II-1) indicates macular atrophy with pigmentation, and OCT displays retinal atrophy without obvious schisis. Note: MS = macular schisis; FS = foveal schisis; FLPS = foveo-lamellar schisis, plus peripheral schisis; PLS = peripheral lamellar schisis; NS = no schisis; SW = Spoke-wheel; ME = macular edema; RPEAM = retinal pigment epithelial alteration or mottling; VAAs = vascular abnormalities; RD = retinal detachment; RA = retinal atrophy.

**FIGURE 4:**
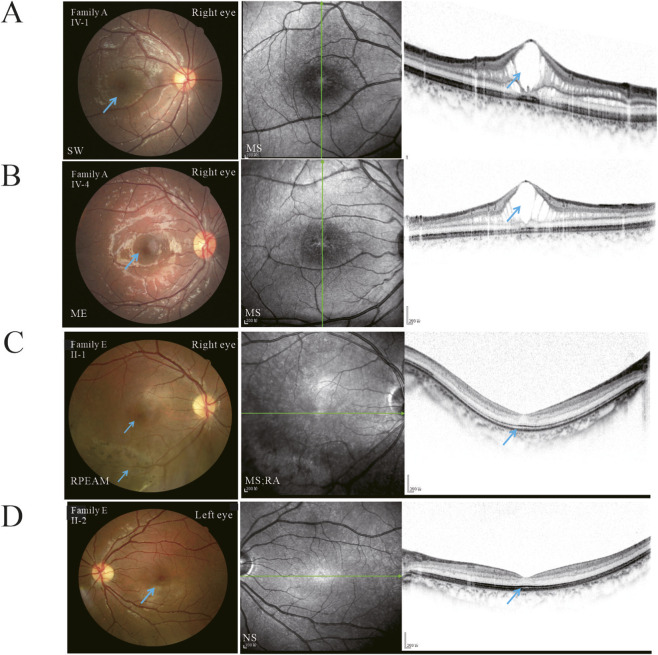
Fundus photographs and optical coherence tomography (OCT) image illustrating retinal abnormalities in X-linked retinoschisis (XLRS) with identical mutations. **(A)** Fundus photographs and OCT of patients with Q43* in Family A IV-4. Fundus check of a 9-year-old revealed scattered clustered pigmentation deposits in the retina, accompanied by spoke-wheel changes and edema in the macular. OCT reveals foveal schisis mainly involving the inner nuclear layer (INL) and the outer nuclear layer (ONL). **(B)** Fundus photographs and OCT of patients with Q43* in Family A IV-1. Fundus examination of a 4-year-old patient displays scattered, clustered pigmentation deposits in the retina and macular edema. OCT indicates foveal schisis involving INL and ONL. **(C)** Fundus photographs and OCT of a patient with R182C in Family E II-1. Fundus examination of a 15-year-old patient displays pigmentary abnormalities in the peripheral retina and relatively normal macula. OCT reveals peripheral retinoschisis only and relatively normal macular structure. **(D)** Fundus photographs and OCT of patients with R182C in Family E II-2. Left eye of a 15-year-old patient with normal fundus and retinal structure. SW = Spoke-wheel; MS = macular schisis; RPEAM = retinal pigment epithelial alteration or mottling; NS = no schisis; RA = retinal atrophy.

Fundus images revealed pigmentary abnormalities (32.26%, 10/31 eyes), macular edema (51.61%, 16/31 eyes), retinal atrophy (16.13%, 5/31 eyes), and spoke-wheel pattern (22.58%, 7/31) (Figure 3). OCT images demonstrated typical macular retinoschisis (MS, n = 27 eyes, 87.1%), peripheral retinoschisis (PS, n = 6 eyes), and atrophic abnormalities (n = 5 eyes). No significant difference was observed in schisis type (macular or peripheral schisis) between group A and group B (*P* = 0.314). Bilateral and symmetric macular retinoschisis was detected in 12 patients (24 eyes), except for Families A II-2, E II-1, II-2, and J II-1. One patient (Family J II-1) exhibited complete retinal atrophy without visible schisis. Two siblings (Families E II-1 and II-2) who harbored R182C exhibited peripheral schisis with or without fovea involvement in one eye and a relatively normal fellow eye (Figure 4).

Schisis cavities, which usually occurred in the macula, were mainly localized in the inner nuclear layer (INL, 87.10%), with variable involvement of the outer nuclear layer (ONL, 83.87%), ganglion cell layer (GCL, 12.90%), and even outer plexiform layer (OPL, 3.23%). No significant correlation was found between the retinal layer involved and mutation type (group A and group B, *P* = 0.99). Schisis changes involved four layers in one eye (3.23%), three layers in three eyes (9.68%), two layers in 22 eyes (70.97%), and one layer in one eye (3.23%) (Figure 3).

Of the 16 patients, complications were varied. RD was identified in two patients, and one patient (the right eye of Family A II-1) suffered cataract and vitreous hemorrhage (VH) based on clinical history. Due to the replacement of the diagnostic equipment during the study period, we analyzed and statistically processed the valid ERG data of 23 eyes of 12 patients. Compared to normal eyes, all affected eyes indicated a reduction in a- and b-wave (*t* = −3.98 *P* = 0.000) amplitudes (*t* = −7.19 *P* = 0.000) and the b/a ratio (*t* = −4.149 *P* = 0.000). Two patients had the c.127C>T p.Q43* mutation and exhibited mild DA 10.0 ERG, even accompanied by a preserved b-to-a amplitude ratio (b/a = 1.72; b/a = 1.63) (Supplementary Figure S1).

#### Rare XLRS phenotypes

We conducted a detailed analysis to further investigate the relation between clinical features and the genetic variants found in our study (Figure 1). The results illustrated that individuals with the same mutation exhibited various clinical features and structural and functional abnormalities (Families A IV-1, A IV-4, D II-1, E II-1, and E II-2). Notably, almost all patients with the Q43* variant exhibited scattered fundus pigmentation, except for Family A II-2, who presented with severe retinal atrophy. Due to the patient’s intolerance to fundus photography, relevant data were obtained via direct fundus examination and are therefore not illustrated (Supplementary Figure S2). Moreover, two patients harboring the R182C variant both demonstrated exotropia and similar macular findings with or without peripheral schisis in one eye, and the contralateral eyes appeared anatomically normal (Figure 3, (Supplementary Figure S3). However, despite sharing an identical Q43* mutation, patient in family A II-2 had more noticeable retinal atrophy and pigment abnormalities in his eyes, whilst other individuals (IV-1, IV-4) had more noticeable macular splitting. Similar variability was noted in families, family D II-1’s OCT displayed macular splitting, while the case family A II-2 with the identical mutation manifested retinal macular and peripheral schisis. Additionally, in line with previous literature, the ocular phenotypes among the carriers (n = 9) in this cohort were largely normal (Supplementary Figures S4 and S5).

## Discussion

Herein, we identified 12 distinct variants in a cohort of 16 patients from 13 unrelated Chinese families, including five novel mutations, consistent with previous large-scale studies (Pennesi et al., 2018; Orès et al., 2018). Our results indicated that most mutations were localized within the discoidin domain, with missense mutations being the predominant type, which is consistent with previous reports (Taku Wakabayashi et al., 2023; Lee et al., 2025). With our cohort, the prevalence of nonsense mutations (3/12), which detected in the northern Chinese population, was higher than in European population, suggesting potential ethnic variations worthy of larger validation studies. Otherwise, large insertions involving exon three were less frequent than previously reported (Lee et al., 2025). These findings underscore the ethnic variability of *RS1* mutations, warranting further investigation.

Age and visual acuity did not statistically correlate, consistent with previous studies (Menke et al., 2011; Orès et al., 2018). Furthermore, consistent with previous research, the mean VA of patients with an average age of 11.2 years was 0.58 Log MAR (Hahn et al., 2022). While Tropicamide was used for cycloplegia to balance efficacy with participant safety and feasibility, we acknowledge that agents such as cyclopentolate provide a deeper cycloplegic effect. However, the protocol used is considered sufficient for obtaining reliable baseline refraction in the context of this study. Earlier genotype-phenotype studies have demonstrated that null mutations have worse VA than missense mutations (Vincent et al., 2013; Hahn et al., 2022). However, we did not observe any significant difference between groups A and B based on the visual acuity, which is consistent with the results of Vincent et al. (Fahim et al., 2017). Generally, Fenner and Leo’s team observed that VA often exhibits a modest increase over the initial 2 decades of life, serving as a primary trial endpoint and highlighting the significance of patient selection for potential treatment trials (Beau et al., 2023; Hahn et al., 2022). Nonetheless, our findings revealed no difference in the mean logMAR BCVA between patients under 10 and those over 10 years old. This emerging trend warrants further investigation and should be considered when assessing VA progression in future natural history and the optimal intervention window for therapeutic studies on XLRS. It is imperative to enlarge the sample size and perform a longitudinal study of VA.

Despite occasional bilateral and asymmetric changes, the funduscopic findings (pigmentary abnormalities, macular edema, retinal atrophy, spoke-wheel patterns) in our study were predominantly consistent with previously published data (Gao et al., 2021; Hahn et al., 2022). The identification of five novel *RS1* mutations, expands the mutational spectrum of XLRS. Meanwhlie, given the small sample size in this study, the distinct pigment clusters in patients with specific *RS1* mutations (Q43*) should be considered preliminary, which may offers a potential diagnostic marker for this specific genotype, further validation should be confirmed. What’s more, Our observation also corroborate that XLRS exhibits pronounced intra and inter-familial variability even with identical mutations, multicenter studies need to explore potential modifying factors, such as skewed x-chromosome inactivation, environmental influences or genetic background effects (Furlan and Galupa, 2022; Topa et al., 2024; Liu X et al., 2024). The observation of partial retinal function preservation in Q43* carriers, despite the truncating nature of the mutation, challenges conventional assumptions about genotype-phenotype correlations in XLRS and suggests that residual protein function or compensatory mechanisms may play a role in disease modulation (Schooneveld, 2022). However, larger samples are required for additional verification. The progression of X-linked juvenile retinoschisis (XLRS) in this northern Chinese cohort revealed striking age-dependent complications, with RD present in approximately 5.5%–16% of patients with XLRS, which was consistent with previous study (Taku Wakabayashi et al., 2023; Beau et al., 2023). Despite the common belief that PS exacerbates complications, our data revealed no differences between patients with and without PS, potentially due to their young age (Jrvinen et al., 2025).

OCT plays an important role in diagnosis. It revealed cystic changes in 87.1% of eyes, predominantly in the inner and outer nuclear layers, consistent with previous findings, whereas the prevalence of PS at 19.35% was lower than previously reported (43%–79%) (Orès et al., 2018; Hahn et al., 2022). Previous observations indicated that macular atrophy was seen in specific elderly individuals without schisis alterations (Vincent et al., 2013). We reported two patients exhibiting retinal atrophy without distinctive schisis, which was more common in cases with nonsense mutations (families A and J), and the prevalence of atrophy was lower than that described in Taiwanese patients (Wang et al., 2015). Excluding Taku et al. and Pennesi et al., the incidence of schisis may be influenced by the age of distribution of the cohort, and schisis cavities occasionally underwent reabsorption over time (Taku Wakabayashi et al., 2023; Pennesi et al., 2022). Consequently, the macular atrophy was regarded as a presumptive sign of prior schisis, as verified by their previous history in this study. Missense mutations are almost always present in affected individuals with normal macular anatomy, according to research by Lee et al. (2025). Notably, related individuals with R182C exhibited discordant phenotypes between the two eyes: one eye exhibited an anatomically normal fovea in OCT, whereas the other eye presented with macular and peripheral schisis (Family E), corroborating previous literature indicating that such occurrences are exclusive to missense mutations (Rahman et al., 2020). The mechanisms behind the occurrence of different types of schisis remain unclear. Tao Xu et al. demonstrated that retinoschisin as a calcium flux modulator, plays a key role in pineal gland calcification in mice through both extracellular and intracellular pathways (Liu X et al., 2024). The finding of distinct asymmetric schisis appeared consistantly in our limited cohort carrying R182C, we propose that the possible reasons may lie in the distribution and secretion patterns of the retinoschisin protein, however, further studies are mandatory to rigorously assess the sensitivity and specificity. Once these potential phenotypic markers were valided, expands the diagnostic criteria for XLRS, particularly in sporadic cases where unilateral findings might otherwise suggest alternative diagnoses.

Bowles et al. and Vincent et al. revealed that a stronger anticipated impact of the *RS1* mutation (null mutations) on the protein function corresponded with a more significant alteration in the b-to-a ratio (Bowles et al., 2011; Vincent et al., 2013). Bowles et al. reported elevated b-wave amplitude beyond their normal limit of 374 mV for three patients, which is rare in XLRS (Bowles et al., 2011). Notably, for two siblings with the c.127C>T nonsense mutation, we found elevated b-wave amplitudes in response to the DA 10.0 ERG stimulation, accompanied by a preserved b-to-a amplitude ratio. This contrasts sharply with classic XLRS cases, where b-wave amplitudes typically fall below 20 μV with ratios <1.0. The retention of near-normal bipolar cell function suggests that the truncated Q43* protein may retain partial activity through alternative translational mechanisms or interact with compensatory molecular pathways (Schooneveld, 2022; Tao et al., 2023). The stability of b-to-a ratios in some advanced cases challenges traditional prognostic markers and suggests potential residual synaptic function amenable to emerging therapies (Ambrosio et al., 2019). Notably, the consistent absence of clinically significant manifestations among our female carriers corroborates the characteristic subclinical phenotype profile established in prior studies.

This study has some limitations, including the small cohort size, missing data (fundus autofluorescence imaging), and limited availability of longitudinal electroretinography examinations. Particularly, future research should prioritize multicenter collaborations to validate these associations in larger cohorts, particularly focusing on the mechanisms underlying mutation-specific phenotypic variability. Longitudinal studies are warranted to elucidate the natural history of XLRS in patients with these novel variants, while functional investigations could clarify how truncating mutations like Q43* permit residual retinal function. However, given the relative rarity of XLRS, assembling a large study group was challenging.

In conclusion, this study advances the understanding of X-linked juvenile retinoschisis (XLRS) by characterizing novel *RS1* mutations and their associated clinical phenotypes in a northern Chinese cohort. Twelve mutations in *RS1* were identified, including five novel mutations, which expanded the spectrum. Distinct pigment clusters (Q43*) and asymmetric schisis (R182C) appeared consistently within our limited cohort carrying specific mutations, which may potential facilitate the diagnosis of XLRS. These findings expand the mutational spectrum of XLRS while demonstrating that genotype-phenotype correlations can inform prognostic predictions, such as the partial retinal function preservation observed in Q43* carriers. Given a wide spectrum of clinical variability, molecular diagnosis is crucial alongside clinical evaluation for diagnosis. The comprehensive clinical and genetic characterization of XLRS in this extensive cohort can be beneficial for clinicians in diagnosis and clinical counseling.

## Data Availability

The datasets presented in this study can be found in online repositories. The names of the repository/repositories and accession number(s) can be found in the article/Supplementary Material.
