# Supplementary material for: Clinical characterization and molecular analysis of X-linked juvenile retinoschisis in a northern Chinese cohort
Source: Front Genet. 2026 May 14;17:1796743. doi: 10.3389/fgene.2026.1796743 (PMC13215648; doi:10.3389/fgene.2026.1796743)
Supplement: Supplementary file 1 [file DataSheet1.doc]

**Clinical Characterization and Molecular Analysis in X-linked Juvenile Retinoschisis Cohorts in Northern China**

**Supplemental information files**

**Supplemental Figures**

**Supplemental Tables**

**Supplemental Methods**

**Supplemental Figures**

**
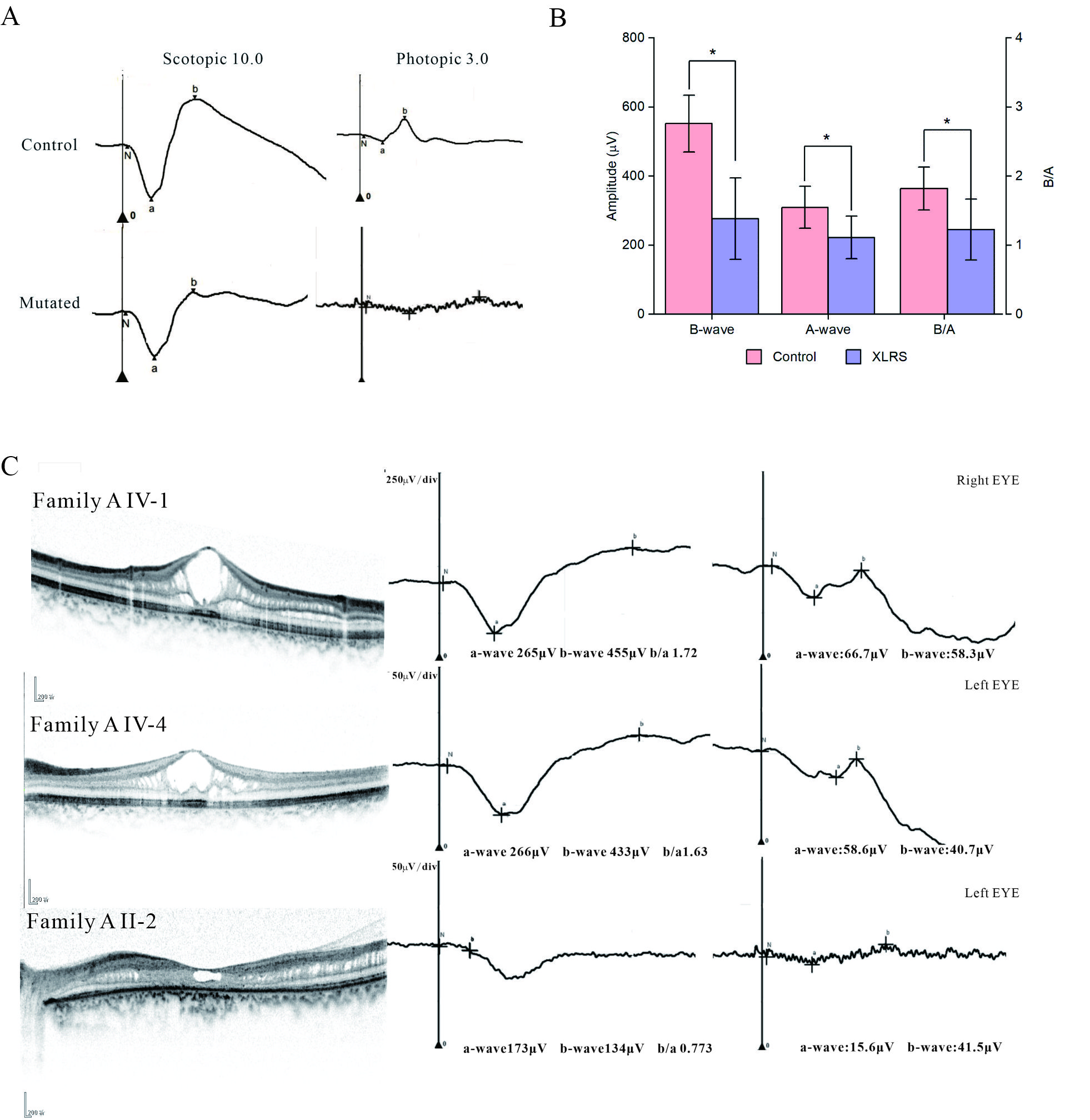
**

**Figure S1**. **Representative electroretinography (ERG) of the affected with Q43* and normal individuals.** (A) The amplitudes of a-wave and b-wave of affected and normal individuals, it responses to scotopic 10.0 rod-specific and photopic 3.0 cone-specific respectively. (B) In rod and cone system, the amplitude of a-wave and b-wave as well as the ratio of B/A were severely reduced (P=0.008; P=0.001; P=0.004). (C) As for family A IV-1, a 4-year-old boy, the amplitudes of a-wave and b-wave in response of scotopic and photopic were 265μV，66.7μV and 455μV, 58.3μV；a 9-year-old boy, the amplitudes of a-wave and b-wave in response of scotopic and photopic were 266μV，58.6μV and433μV, 40.7μV；a 45-year-old male, the amplitudes of a-wave and b-wave in response of scotopic and photopic were 173μV，15.6μV and 134μV, 41.5μV.


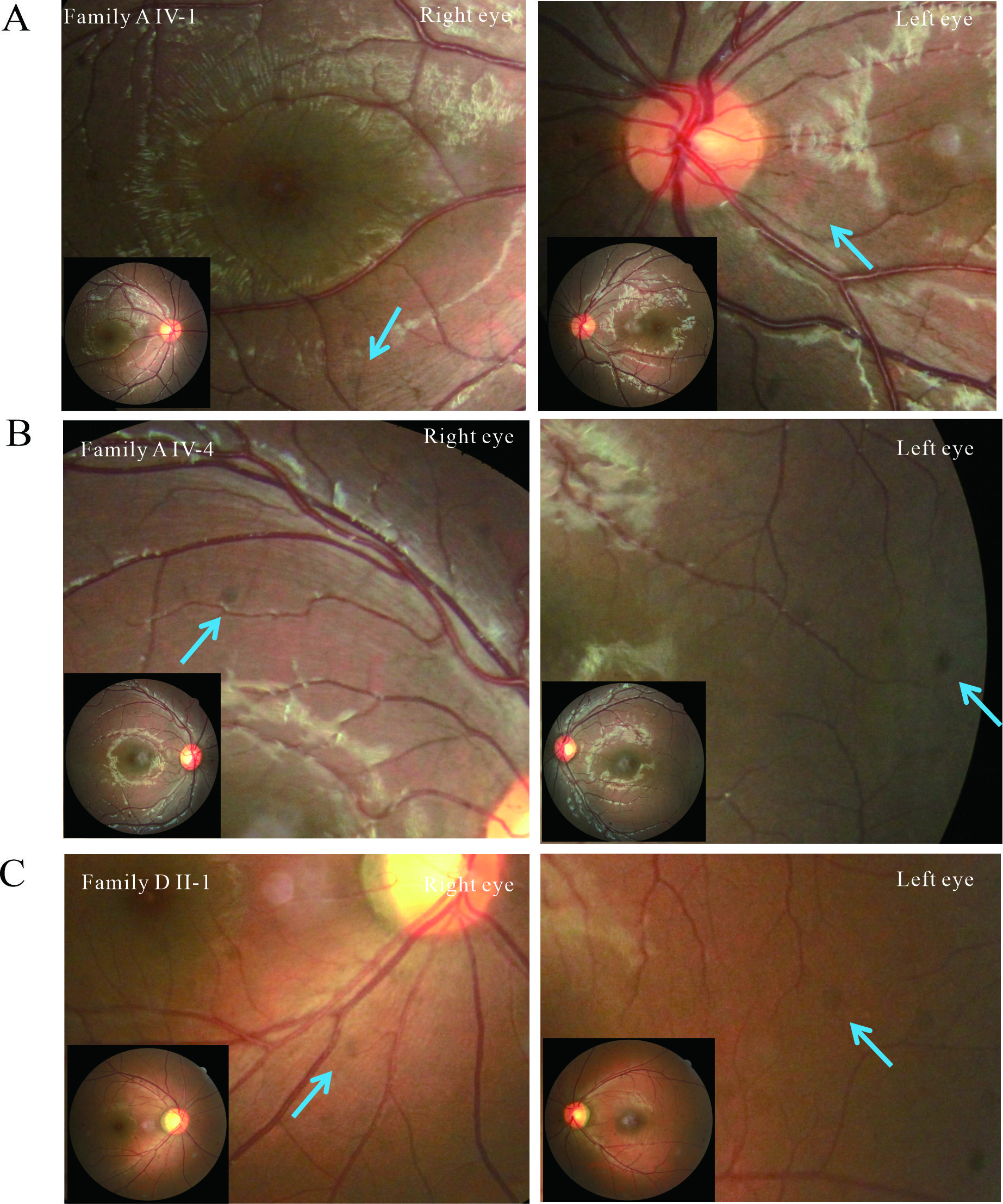


**Figure S2**  **Representative fundus photographs of XLRS patients with Q43***. (A) The fundus photographs of familyA IV-1;(b) The fundus photographs of familyA IV-4;(C) The fundus photographs of familyD II-1;Arrow indicates pigmentation abnormality.


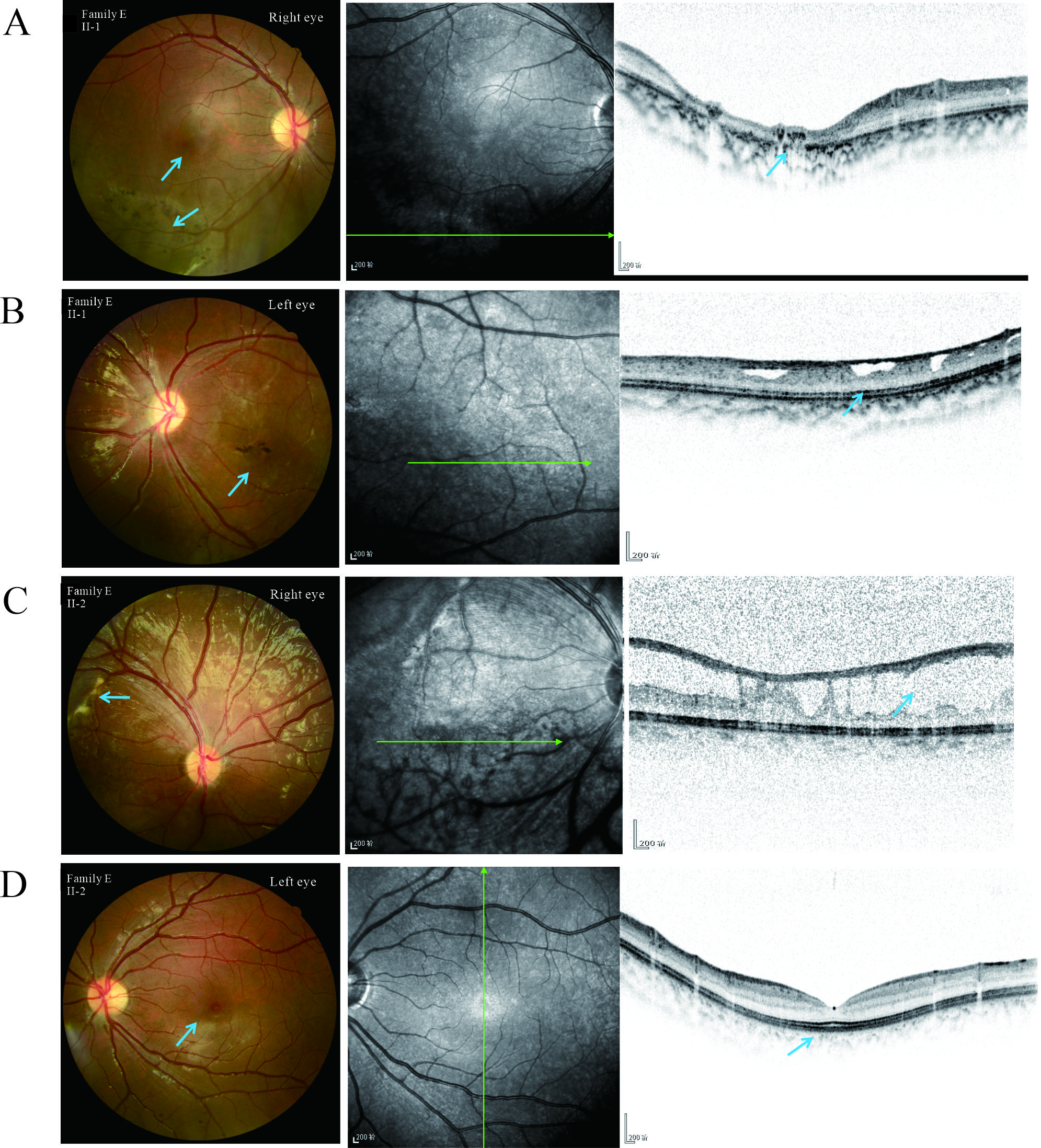


**Figure S3. Fundus photographs and optical coherence tomography (OCT) image showing retinal abnormalities in X-linked retinoschisis with R182C.** (A) Fundus photographs and OCT of right eye of patient (family E II-1) with R182C. (B) Fundus photographs and OCT of left eye of patient (family E II-1) with R182C. (C) Fundus photographs and OCT of right eye of patient (family E II-2) with R182C. (D) Fundus photographs and OCT of left eye of patient (family E II-2) with R182C.

**Figure S4. Fundus photographs and optical coherence tomography (OCT) image of female carriers.**

**
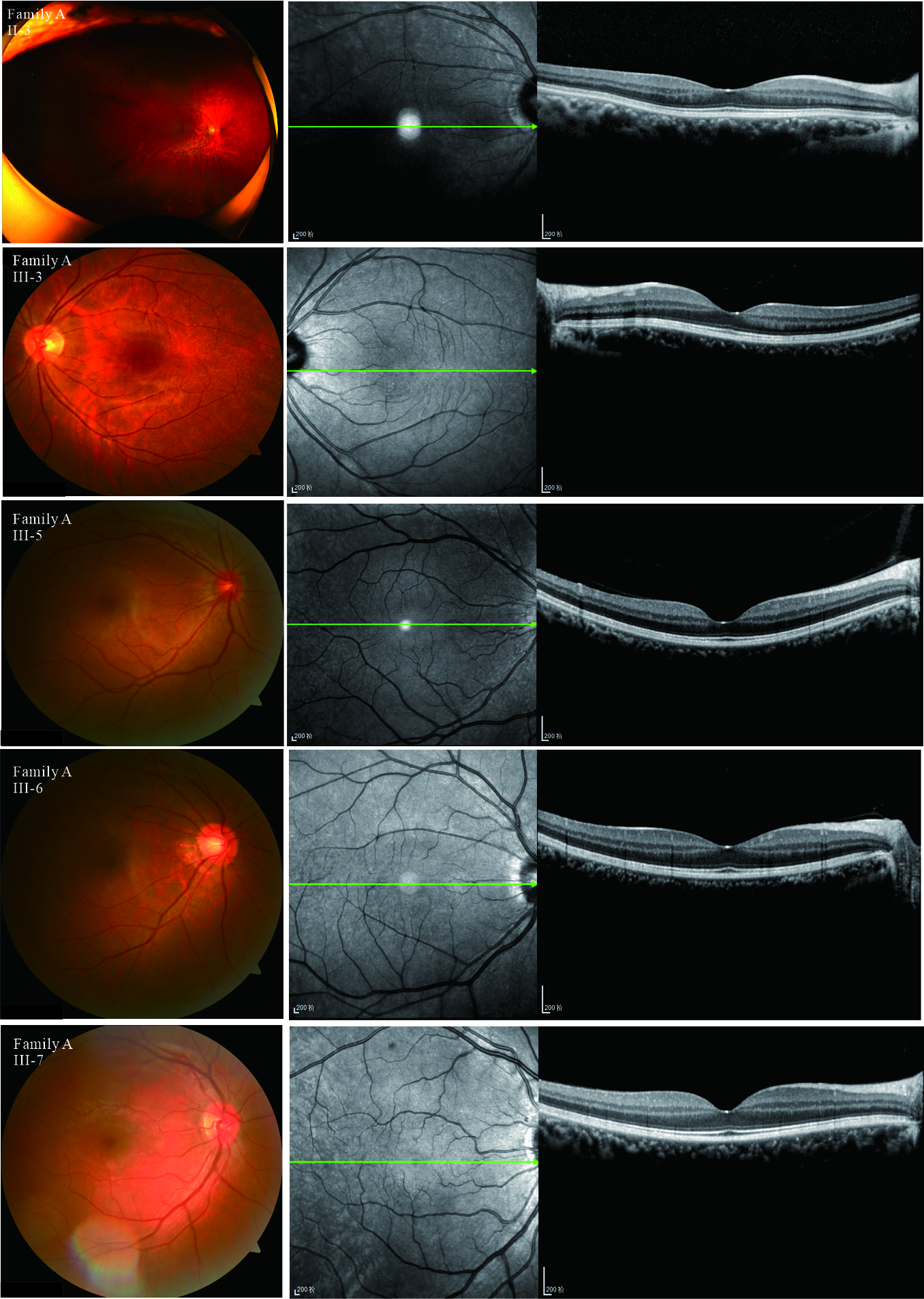
**

**Figure S5. Fundus photographs and optical coherence tomography (OCT) image of female carriers.**

**
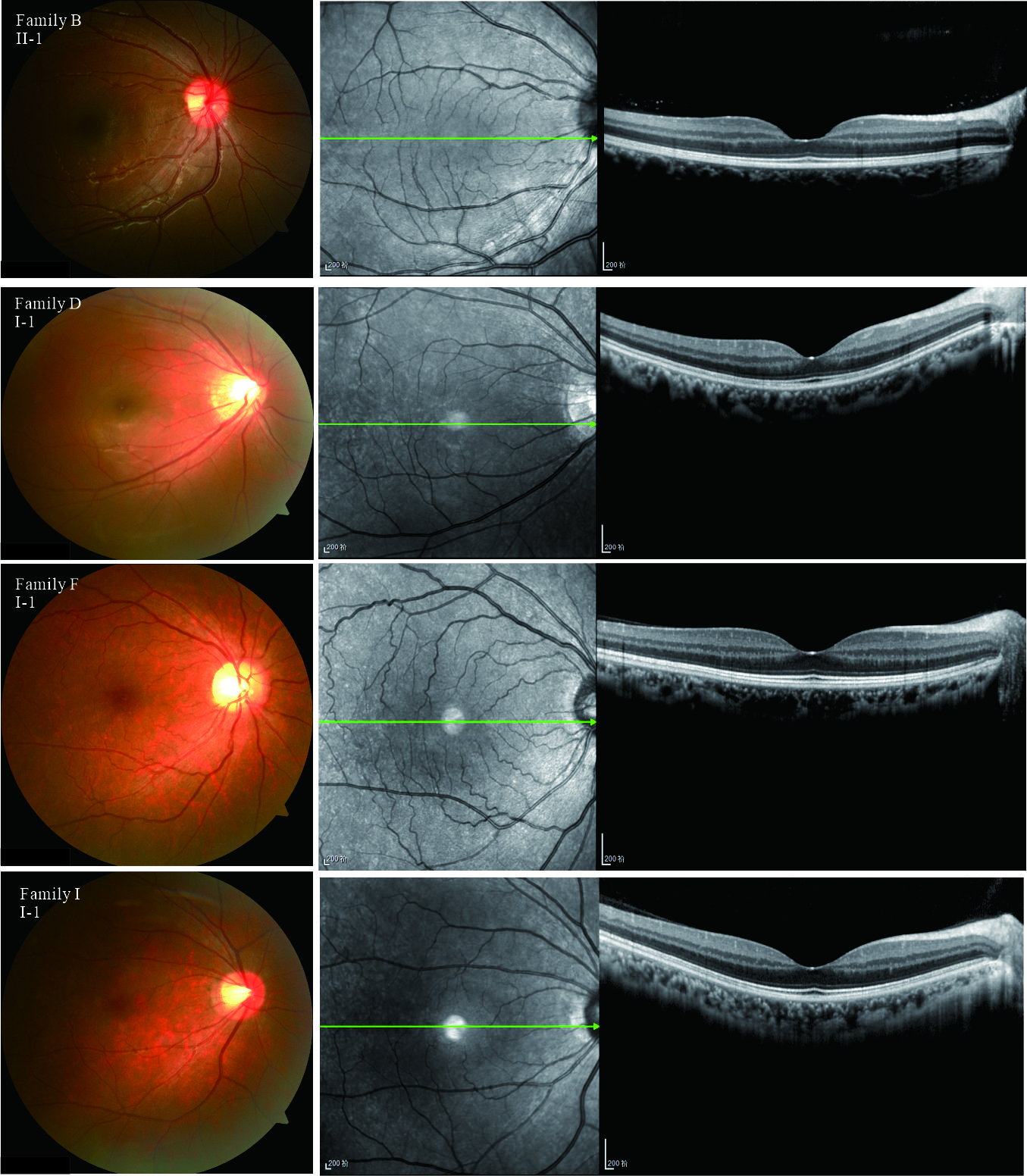
**

**Supplemental table 1.** Summary of primers of *RS1* gene.

Note: F Forward; R Reverse.

| **Exon** | **Direction** | **Primer sequences (5’-3’)** | **Size of amplified fragment (bp)** | **Annealing temperature(℃)** |
| --- | --- | --- | --- | --- |
| 1 | F | GCTCAGCCAAAGACCTAAGA | 350 | 58 |
|  | R | GCCATCCACACAAAGACAAC |
| 2 | F | GTCCCTAGCTTCTTAGCATCTG | 497 | 58 |
|  | R | GGATTACAGGTATGCACCACTAT |
| 3 | F | TGAGTGGTAGCTGTGTGTATTG | 557 | 58 |
|  | R | TGGAGACATGGAGATCTAGGG |
| 4 | F | TCCTTTCTTGCGTGAGTAGTG | 672 | 58 |
|  | R | GGTCTTTCTTCCTTCATCTCTCC |
| 5 | F | AGGGAGAGGGAGAATGAGAT | 430 | 58 |
|  | R | GCTGAAGTTGGTTTGGGATAAG |
| 6 | F | CACCCGCAAACTGCTTTAAC | 604 | 58 |
|  | R | AGACTGCACCTTTCACAGTATC |

**Supplemental table 2** Presumed pathogenic *RS1* variants identified in this program and analysis of the variants by predictive programs.

| **Family No.** | **Group** | **Exon** | **Nucleotide change** | **Protein effect** | **ClinVar Accession** | **Mutation Taster** | **PROVEN** | **Polyphen-2** | **REVEL** | **1000G** | **MAF** | **mutation** | **ACMG** |
| --- | --- | --- | --- | --- | --- | --- | --- | --- | --- | --- | --- | --- | --- |
| A | B | 3 | c.127C>T | p.Q43* | SCV007096142 | DC | - | - | - | - | - | Novel | LP^1^ |
| L | A | 3 | c.176G>A | p.C59Y | SCV007096146 | DC | -3.78 | 1 | 0.754 | - | - | Pubmed:22039241,35456481,22245991 | - |
| G | B | 3 | C.140-141inserTGCTCTG | W50Cfs*38 | SCV007096147 | DC | - | - | - | - | - | Novel | LP^2^ |
| J | B | 4 | c.195T>G | p.Y65* | SCV007096148 | DC | - | - | - | - | - | Novel | LP^3^ |
| F | A | 4 | c.203C>G | p.P68R | SCV007096149 | DC | -7.56 | 0.998 | 0.942 | - | - | Pubmed:30551202,35456481 | - |
| C | A | 4 | c.214G>A | p.E72K | SCV007096150 | DC | -3.648 | 1 | 0.921 | - | 0.00001 | Pubmed:9618178 | - |
| B | B | 4 | c.276G>A | p.W92* | SCV007096151 | DC | - | - | - | - | - | Pubmed:29739629,34645606 | - |
| I | A | 4 | c.276G>T | p.W92C | SCV007096152 | DC | -7.561 | 1 | 0.982 | - | - | Pubmed:28272453 | - |
| H | A | 4 | c.311A>G | p.N104S | SCV007096153 | DC | -4.705 | 1 | 0.921 | - | - | Pubmed:32300273,34645606 | - |
| K | A | 5 | c.512G>A | p.G171E | SCV007096143 | DC | -4.362 | 1 | 0.819 | - | - | Novel | LP^4^ |
| E | A | 6 | c.544C>G | p.R182C | SCV007096144 | DC | -3.864 | 1 | 0.725 | - | - | Novel | LP^5^ |
| M | A | 6 | c.626G>A | p.R209H | SCV007096145 | DC | -0.987 | 1 | 0.796 | - | - | Pubmed:9326935 | - |

Abbreviations: DC Disease causing; N: Not novel. LP:Likely Pathogenic; MAF, Minor allele frequency. Group B including nonsense or insert mutations; LP^1^:PVS1+PM2_Supporting; LP^2^:PVS1+PM2_Supporting; LP^3^:PVS1+PM2_Supporting; LP^4^:PM1+PM5+PM2_Supporting+PP3; LP^5^:PM1+PM5+PM2_Supporting+PP3;

**Supplemental Methods**

1. Fundus Photography (Retinography)

Device: Hotline retinal camera

Manufacturer: Gaoshi Medical Equipment Co.

Country: China

Parameters:

Field of view: 45°

Image resolution: 2048 × 2048 pixels

Illumination: white LED

Pupillary dilation: tropicamide (minimum pupil diameter 6 mm)

Exposure: auto mode, manually adjusted if needed

Quality check: no motion artifact, proper foveal centration

2. Full-Field Electroretinography (ERG)

Device: ROLAND CONSULT RETI-PORT/SCAN 21

Manufacturer: Roland Consult GmbH

Country: Germany

Procedures:

Performed in accordance with ISCEV standards (Robson et al., 2022).

Dark adaptation: 20 min

Light adaptation: 10 min

Electrodes: DTL fiber active electrode; reference at outer canthus; ground on the forehead

Sampling rate: 1,000 Hz

Bandwidth: 0.3–300 Hz

Averaging: ≥3 traces per stimulus

Quality control: baseline noise <10 µV

Stimulus conditions:

Scotopic rod response (0.01 cd·s/m²)

Scotopic maximal response (3.0 cd·s/m²)

Photopic single-flash response (3.0 cd·s/m², background 30 cd/m²)

30-Hz flicker ERG (3.0 cd·s/m²)

3. Optical Coherence Tomography (OCT)

Device: Spectral-Domain OCT (Spec-TR-04852)

Manufacturer: Heidelberg Engineering GmbH

Country: Germany

Acquisition parameters:

Scan protocol: Horizontal macular cube

Number of B-scans: 25–49

Axial resolution: ~7 µm

Transverse resolution: ~14 µm

Scan length: 6 mm

ART averaging: 5–15 frames

Segmentation: Automated with manual correction

Minimum acceptable signal: ≥20 dB

Time-domain OCT (historic data): resolution 10–12 µm, scan length 6 mm

Quality Control Procedures:

All imaging and ERG were performed by two senior ophthalmologists.

Images were independently reviewed by two retina specialists.

OCT B-scan segmentation was corrected manually when necessary.

ERG recordings were repeated if noise exceeded the threshold.
